# Correlation of Childhood Psychological Abuse and Neglect With Mental Health in Chinese College Students During the COVID-19 Pandemic

**DOI:** 10.3389/fpsyt.2021.770201

**Published:** 2022-01-05

**Authors:** Zhaohong Zhu, Pu Li, Luyao Hao

**Affiliations:** School of Exercise and Health Sciences, Xi'an Physical Education University, Xi'an, China

**Keywords:** psychological abuse and neglect, mental health, COVID-19, childhood maltreatment, college students

## Abstract

Experience of childhood maltreatment is a major factor affecting adult mental health. The purpose of this study was to understand the association of childhood psychological abuse and neglect with mental health in college students during the COVID-19 pandemic. An online questionnaire survey was conducted from February 21 to March 12, 2020. The participants were 200 students at a university of physical education in Shaanxi Province, China. Participants completed the Child Psychological Abuse and Neglect Scale and the Mental Health Self-Report Questionnaire. Regarding childhood maltreatment experience, 52.5% of respondents screened positive for childhood psychological abuse, 55.8% for psychological neglect, and 43.6% for both. Moreover, 37.6% of participants screened positive for psychological health problems during the pandemic. Childhood psychological abuse and neglect were positively associated with mental health problems during the COVID-19 pandemic. A regression analysis revealed that the reproving dimension of psychological abuse was a risk factor for mental health problems in college students during the COVID-19 pandemic.

## Introduction

To stop the spread of COVID-19, most of the world's governments, including that of China, adopted unprecedented social isolation measures, such as lockdowns, minimizing outings, social distancing, and canceling or minimizing meetings (1). These measures undoubtedly impacted the psychological state of various populations. The unpredictability and uncertainty of life, as well as economic changes associated with the COVID-19 pandemic are major stressors that have led to psychological distress, with college students at a higher risk (2). College students may also have struggled with loneliness, isolation, and severe psychological distress during the pandemic due to school closures and alienation from classmates and friends (3). Although the vast majority of colleges and universities have adopted measures, such as online learning and tutors to guide classes remotely, psychological interventions to popularize epidemic knowledge, and other measures to relieve students' psychological pressure and maintain a normal school life (4), many students have experienced varying degrees of psychological problems after the resumption of school (5). This has seriously affected the physical and mental health of college students. Therefore, it is especially important to identify factors that might exacerbate college students' psychological problems in response to stressful events, such as childhood maltreatment.

Child maltreatment has long-term negative effects on the physical and mental health of individuals, and is one of the main risk factors leading to psychological problems in adolescents (6). It is thus reasonable to consider that child maltreatment may increase the probability of psychological problems during stressful events in college students. Since the 1960s, child maltreatment has become a worldwide public health problem. Child maltreatment, including abuse (physical, psychological, and sexual) and neglect (psychological and physical), is one of the world's most troubling problems (7, 8). Psychological abuse and neglect are the core issues of childhood maltreatment, which refers to continuous, repeated inappropriate behavior toward a child for which one has responsibilities and obligations and/or to which one is close. Some examples of maltreatment include intimidation, abasement, interference, indulgence, and emotional neglect (9). Psychological abuse and neglect are among the strongest predictors of psychological problems, more so than physical or sexual abuse (6).

According to Young's schema theory (10), psychological abuse and neglect are closely related to the schemas of a loss of self-worth (such as emotional deprivation and cognition of social isolation), which can cause the development of negative internal work patterns toward themselves, and thus have lasting harmful effects on an individual's physical and mental health (6). In line with this hypothesis, it has been found that psychological abuse and neglect increase negative emotional regulation and coping strategies (11)—such as emotional inhibition and rumination, which are predisposing factors for depression (12)—as well as emotional inhibition and avoidance strategies (13, 14).

These studies indicate that psychological abuse and neglect may be risk factors for a variety of psychological problems. However, it should be noted that most of these studies have focused on psychological disorders in adulthood, and few have focused on psychological problems during stressful events. Although psychological abuse is known to bring greater risks to the development of young people, not all victims of childhood abuse exhibit more psychological problems in adolescence. Indeed, a considerable number of individuals still achieve healthy development after experiencing psychological abuse (15). Therefore, it is particularly important to understand the impact of psychological abuse experience on the occurrence of psychological problems during the epidemic, and to provide effective psychological guidance and intervention strategies for college students in future public health emergencies. Based on the above theories and empirical research, we proposed the following two hypotheses: (1) Childhood abuse and neglect are positively correlated with mental health problems during the epidemic; (2) Psychological abuse and neglect can predict the incidence of psychological problems during the epidemic.

## Materials and Methods

### Participants

The study's participants were college students at a university of physical education in Shaanxi Province, China, all of whom were required to isolate at home from February 21 to March 12, 2020. A questionnaire was created on Wenjuanxing (www.wjx.cn), an online survey platform. Adopting convenience sampling, the questionnaire link was distributed through the class WeChat group of the respondents on campus. Students volunteered to participate in the survey and filled in the questionnaire by clicking the link or scanning the two-dimensional code (system lucky draw after submission of Wenjuanxing). The questionnaire was anonymous and all stored data were confidential. A total of 200 questionnaires were returned. After removing questionnaires with missing and/or invariable responses, 181 valid questionnaires remained. The average age of participants was 21.32 (±1.311) years. Of the final sample, 65 respondents (35.9%) were male and 116 (64.1%) were female; 27 (14.9%) were freshmen, 18 (9.9%) sophomores, 38 (21.0%) juniors, 78 (43.1%) seniors, and 20 (11.0%) postgraduates; 44 (24.3%) majored in physical education and 137 (75.7%) majored in other subjects.

### Sample Size Adequacy

A *post-hoc* power analysis using G*Power version 3.1.9.7 (Franz Faul, Kiel University, Germany) was used to determine the adequacy of the sample size (16). Using the medium effect size (*D* = 0.3, α = 0.05), for a sample size of 181, the statistical efficacy (1-ß) obtained estimated to test the hypotheses of this study was 0.99, thus justifying the adequacy of our sample size.

### Measurement Instruments

#### The Child Psychological Abuse and Neglect Scale

This self-report questionnaire was developed by Deng et al. (17) as a retrospective measurement instrument applicable to Chinese people. It mainly investigates the psychological abuse experience of an individual in childhood (younger than 18 years), including family abuse, neglect, and the ways in which guardians treated them. It contains 31 items, each rated on a five-point (0–4) scale. The two subscales, respectively, assess psychological abuse and psychological neglect. The psychological abuse subscale contains 14 items in the three dimensions of reproving, intimidation, and interference. The sum of the scores of the three dimensions is used to measure psychological abuse, with a higher total score indicating more severe abuse. The psychological neglect subscale contains 17 items that assess the three dimensions of emotional neglect, educational neglect, and physical neglect. The sum of the scores for these three dimensions is used to measure neglect-maltreatment, with a higher total score indicating more severe neglect-maltreatment. The quotient of the total score and the number of items in a subscale is defined as the factor score. A factor score ≥1 is considered positive (17). The test-retest reliability values of the overall scale, psychological abuse subscale, and psychological neglect subscale were 0.82, 0.80, and 0.76, respectively (18).

#### The Mental Health Self-Report Questionnaire

The Mental Health Self-Report Questionnaire [SRQ-20; (19)] contains 20 “yes” or “no” questions. A response of “yes” is scored as 1, and a response of “no” as 0; thus, the highest possible score is 20. This questionnaire primarily screens for common post-disaster psychological reactions, such as depression, anxiety, and physical discomfort, with a higher score indicating more severe psychological problems. The reference cut-off score indicating the need for clinical intervention is 7 [i.e., care should be given to respondents scoring higher than 7; (19)]. The split-half reliability of the questionnaire is 0.748, the Cronbach's alpha reliability coefficient of each item is 0.778–0.789, and the Cronbach's alpha reliability coefficient of the overall questionnaire is 0.792 (20). A comprehensive analysis of the reliability of the SRQ-20 is provided in the user's guide to the SRQ-20 issued by the WHO (19).

### Procedure

Approval from the ethics committee of Xi'an Physical Education University was received before the study began. Respondents provided online informed consent online before completing the questionnaire. All participants were told that their privacy would be protected and that they were free to withdraw from the study at any time. The anonymity of the study was also emphasized before data collection. In view of the fact that students were taking online classes during the questionnaire distribution period, the second author distributed the questionnaires through the class WeChat group with the help of the monitor or the teacher.

### Data Analysis

Collecting data via a self-report questionnaire introduced the possibility of common method errors. Therefore, respondents were reassured that their data would be anonymous. The statistical analysis was carried out using SPSS version 22.0 (IBM Corp., Armonk, NY, United States). Before statistical analysis, the skewness and kurtosis in the frequency statistics were used to test the normal distribution of the data. The skewness and kurtosis of all variable data were <1, which indicates that the data had an approximately normal distribution. The subsequent data analysis was carried out in three steps. First, descriptive statistics were used to present demographic data. The prevalence rate of child abuse was calculated according to the cut-off score adopted by Deng et al., including a mean score of psychological abuse ≥1 and a mean score of neglect ≥1 (17). The detection rate of psychological problems among college students during the COVID-19 pandemic was calculated according to the WHO cut-off score of the SRQ-20 (19). Second, a correlation analysis was performed to explore the relationship between psychological abuse and neglect and psychological problems during the COVID-19 pandemic. Third, a binary logistic regression analysis was performed to obtain odds ratios (OR) and 95% confidence intervals (CIs).

## Results

### Demographic Characteristics of Participants

A participant was considered to have experienced childhood psychological abuse or neglect if their score averaged ≥1 on the respective subscales. On this basis, 95 (52.5%) screened positive for psychological abuse, 101 (55.8%) for neglect, and 79 (43.6%) for both. According to the SRQ-20 scores, 68 (37.6%) of respondents screened positive for psychological problems during the COVID-19 pandemic. Table 1 shows the results for childhood trauma and the SRQ-20 scores of participants according to their different demographic characteristics.

**Table 1:** Differences in demographic variables of childhood trauma and the SRQ-20 scores.

**Variable**		***n* (%)**	**Reproving**	**Intimidation**	**Interference**	**Emotional neglect**	**Educational neglect**	**Physical neglect**	**The SRQ-20 scores**
**Grade**	Freshmen	27 (14.40)	1.80 ± 1.01	1.67 ± 1.01	1.25 ± 0.84	1.54 ± 0.68	1.61 ± 1.07	1.36 ± 0.73	8.44 ± 5.409
	Sophomore	18 (9.90)	1.10 ± 0.69	1.22 ± 0.98	0.57 ± 0.72	1.37 ± 0.74	1.42 ± 0.89	1.10 ± 0.88	5.33 ± 5.369
	Junior	38 (21.00)	1.18 ± 1.08	1.11 ± 0.89	0.781 ± 0.74	1.13 ± 0.76	1.13 ± 0.95	0.88 ± 0.81	5.34 ± 5.649
	Senior	78 (43.10)	1.41 ± 1.05	1.41 ± 0.88	0.88 ± 0.77	1.25 ± 0.78	1.21 ± 1.07	1.00 ± 0.90	6.73 ± 5.419
	Postgraduate	20 (11.60)	1.35 ± 1.01	1.20 ± 0.62	1.04 ± 0.62	1.16 ± 0.67	1.19 ± 0.74	1.06 ± 0.72	5.75 ± 5.056
*F*			1.86	1.90	2.71^*^	1.49	1.20	1.38	1.635
*p*			0.12	0.11	0.03	0.21	0.31	0.24	0.167
**Gender**	Male	65 (36.50)	1.49 ± 1.05	1.38 ± 0.87	0.87 ± 0.79	1.41 ± 0.73	1.26 ± 1.02	1.17 ± 0.83	6.770 ± 5.545
	Female	116 (63.50)	1.32 ± 1.01	1.32 ± 0.92	0.92 ± 0.76	1.19 ± 0.75	1.28 ± 1.00	0.98 ± 0.85	6.27 ± 5.432
*t*			1.08	0.43	−0.39	1.96^*^	−0.12	1.42	0.592
*p*			0.28	0.67	0.70	0.05	0.91	0.16	0.555
**Major**	PE major	44 (24.30)	1.36 ± 1.04	1.43 ± 0.90	0.89 ± 0.72	1.30 ± 0.84	1.28 ± 1.01	1.11 ± 0.91	7.360 ± 6.329
	Non-PE major	137 (75.70)	1.39 ± 1.03	1.31 ± 0.90	0.91 ± 0.78	1.26 ± 0.72	1.27 ± 0.99	1.03 ± 0.82	6.150 ± 5.145
*t*			−0.14	0.76	−0.11	0.36	0.07	0.60	0.285
*p*			0.89	0.45	0.91	0.72	0.95	0.55	0.202

* *p < 0.05*.

Descriptive statistics for all variables are presented in Table 1. There were differences in the interference dimension between different grades. Specifically, there were significant differences between freshmen and sophomores, and between juniors and seniors (*F* = 2.71, *p* = 0.03). *Post-hoc* comparisons showed that freshman students had a significantly higher interference dimension score (1.25 ± 0.84) than sophomore (0.57 ± 0.72, *p* < 0.01), junior (0.78 ± 0.74, *p* = 0.02) and senior (0.88 ± 0.77, *p* = 0.03) students.

We compared the psychological conditions of students with and without psychological abuse and psychological neglect during the epidemic. The results revealed significantly higher SRQ-20 scores indicating more severe psychological problems in students with psychological abuse and psychological neglect (in the Supplementary Table 1).

### Correlations Between Childhood Trauma and the SRQ-20 Scores

The correlations between childhood trauma and the SRQ-20 scores during the COVID-19 pandemic were analyzed. As shown in Table 2, the SRQ-20 scores were positively correlated with the dimensions of psychological abuse and neglect. Specifically, the SRQ-20 scores was positively related to reproving (*r* = 0.57, *p* < 0.01), intimidation (*r* = 0.52, *p* < 0.01), interference (*r* = 0.43, *p* < 0.01), emotional neglect (*r* = 0.44, *p* < 0.01), educational neglect (*r* = 0.42, *p* < 0.01), physical neglect (*r* = 0.36, *p* < 0.01). Correlation analysis showed that higher scores in the dimensions of psychological abuse and neglect were associated with more severe psychological problems.

**Table 2:** Correlation between childhood trauma and the SRQ-20 scores.

	**Reproving**	**Intimidation**	**Interference**	**Emotional neglect**	**Educational neglect**	**Physical neglect**
The SRQ-20 scores	0.57^**^	0.52^**^	0.43^**^	0.44^**^	0.42^**^	0.36^**^

** *p < 0.01*.

In addition, the results of the correlations between the dimensions of childhood trauma were presented in the Supplementary Table 2. Correlation analysis showed reproving was positively related to intimidation (*r* = 0.82, *p* < 0.01), interference (*r* = 0.69, *p* < 0.01), emotional neglect (*r* = 0.72, *p* < 0.01), educational neglect (*r* = 0.62, *p* < 0.01) and physical neglect (*r* = 0.59, *p* < 0.01). Intimidation was positively related to interference (*r* = 0.63, *p* < 0.01), emotional neglect (*r* = 0.69, *p* < 0.01), educational neglect (*r* = 0.58, *p* < 0.01) and physical neglect (*r* = 0.52, *p* < 0.01). Interference was positively related to emotional neglect (*r* = 0.53, *p* < 0.01), educational neglect (*r* = 0.49, *p* < 0.01) and physical neglect (*r* = 0.40, *p* < 0.01). Emotional neglect was positively related to educational neglect (*r* = 0.82, *p* < 0.01) and physical neglect (*r* = 0.77, *p* < 0.01). Educational neglect was positively related to physical neglect (*r* = 0.71, *p* < 0.01).

### Effect of Childhood Maltreatment Experience on the SRQ-20 Scores

Psychological problems were taken as the dependent variable (the SRQ-20 score ≥7 indicates psychological problems, and the assigned value was 1; the SRQ-20 score <7 indicates no psychological problems, and the assigned value was 0), and the dimensions of psychological abuse and neglect were taken as independent variables. The binary logistic regression analysis showed that the reproving dimension of psychological abuse (β = 0.78, OR = 2.19, 95%CI = 1.11–4.30, *p* = 0.02) was a risk factor for psychological problems in college students during the COVID-19 pandemic in Table 3.

**Table 3:** Logistic regression analysis of childhood abuse experience and the SRQ-20 scores.

	**β**	**S.E**.	**Wals**	***P-*value**	**OR**	**95%CI**
Reproving	0.78	0.35	5.14	0.02	2.19	1.11–4.30
Intimidation	0.39	0.36	1.19	0.28	1.48	0.73–2.96
Interference	−0.08	0.32	0.06	0.81	0.92	0.49–1.74
Emotional neglect	0.32	0.50	0.40	0.53	1.38	0.51–3.68
Educational neglect	0.19	0.31	0.37	0.54	1.20	0.66–2.19
Physical neglect	−0.21	0.33	0.38	0.54	0.81	0.42–1.57
Constant	−2.11	0.40	27.28	<0.01	0.12	

In addition, the results of multicollinearity analysis were presented in the Supplementary Table 3. The results showed that there is no collinearity problem [tolerance (TOL): reproving: 0.25, intimidation:0.30, interference:0.51, emotional neglect:0.21, educational neglect:0.31, physical neglect:0.38; variance inflation factor (VIF): reproving:4.07, intimidation:3.32, interference:1.96, emotional neglect:4.84, educational neglect:3.26, physical neglect:2.61].

## Discussion

The COVID-19 pandemic has affected the mental health of different populations to varying degrees (2). However, previous studies have mainly focused on the effect of external factors, such as concerns about COVID-19 infection, delayed graduation, employment prospects (5), the amount of negative information received during the epidemic, gender, and place of birth (21). Few studies have examined the effects of internal psychological factors on mental health, such as whether psychological abuse and neglect increase the psychological problems of college students during stressful events. Therefore, this study examined the association of psychological abuse and neglect with mental health in college students during the COVID-19 pandemic. As expected, childhood abuse and neglect were predictive of the incidence of psychological problems during the pandemic. These results extend our understanding of the factors that influence psychological problems in college students during stressful events.

The dimensions of psychological abuse and neglect in this study were positively associated with the psychological problems of college students during the COVID-19 epidemic. Specifically, the higher the degree of child abuse, the higher the incidence of psychological problems when exposed to stressful situations. This result is consistent with previous findings that people with childhood abuse experience are more likely to show various psychological problems when facing stressful events in adulthood (22, 23), and even post-traumatic stress disorder (11). This result can be explained by the theory of helplessness (24). Namely, when people repeatedly suffer from psychological abuse and neglect during childhood, various needs remain unmet and they feel unable to control their environment, leading to a sense of helplessness. This increases the susceptibility to depression (25) and anxiety (14). Furthermore, there is already strong evidence that child maltreatment can lead to abnormal changes in the cortisol response and differences in the morphology of the hippocampus, amygdala, and prefrontal lobe, thereby increasing the possibility of depression (26). Thus, the experience of child maltreatment may increase an individual's susceptibility to stress during public health events, thereby affecting mental health.

This study also identified the risk factors of psychological abuse and neglect that are more likely to cause psychological problems. Scolding in psychological abuse may increase the risk of psychological problems. Scolding is mainly based on verbal humiliation (9). The results of two studies have indicated that high school students who have experienced humiliation are more likely to have physical symptoms and exhibit compulsive behaviors than students with no such experience. Interpersonal sensitivity, depression, anxiety, hostility, phobia, paranoia, psychotic symptoms (13), and the scores of each symptom factor tend to increase with the degree of humiliation (27). Furthermore, as a bad parenting method, scolding can also cause estrangement from parents (28). Another large-scale study conducted in five provinces in China showed that alienation from parents is associated with an increased risk of psychological problems in college students during the COVID-19 pandemic (29). In the present study, only those who experienced scolding had a higher risk of psychological problems during stressful events. A study have pointed out that scolding is negatively correlated with more positive personalities than other forms of psychological abuse and neglect, and positively correlated with negative personalities (30). Long-term experience of negative experiences, such as scolding, humiliation, and a lack of warmth and family security, can lead to low self-esteem and a tendency to develop negative personality traits in adulthood, such as introversion, depression, anxiety, a closed personality, and unhealthy interpersonal relationships. Child with dominant negative personalities grew up in an environment in which they were often scolded, lacked self-confidence, and tended to be timid and hesitant, showing a closed personality (30).

## Strengths and Limitations

Good mental health quality is particularly important in the face of stressful events such as the COVID-19 pandemic. Although schools and relevant departments have provided various preventive measures and policies, the incidence of psychological problems among college students is still worrying, and researchers should address the issues of psychological prevention and control. The effects of psychological abuse and neglect during childhood sometimes only become apparent later in life, such as mental health issues in college students during stressful events. Therefore, identifying the inherent risk factors that affect college students in the face of stressful events provides useful information for improving the response strategies for stressful public health events, identifying high-risk students, and developing psychological crisis interventions for specific populations.

Our research also has some limitations. First, the sample size of this survey was small, and the sample was limited to specific groups from sports colleges. Moreover, because this survey explicitly assessed childhood experiences, there may have been memory and information biases, so the sample may be underrepresented. In addition, strong correlations between variables were found in this study, but only one variable showed significance in the binary regression analysis, after which we conducted a multicollinearity analysis. The results showed that tolerance (TOL) was >0.1 and variance inflation factor (VIF) was <10, therefore we assumed that the problem of collinearity could be ignored (31, 32). Concerning the inconsistency between the correlation analysis and regression analysis, we believe that it may be related to the insufficient sample size. Future research should expand the sample size. Second, we adopted a cross-sectional design, which means that a causal relationship cannot be inferred; longitudinal studies or intervention experiments are needed to better test causality. Third, this study only collected data from Chinese college students, and adopted a rating scale for psychological abuse and neglect with Chinese characteristics. However, psychological abuse and neglect are deeply embedded in the cultural framework of different countries, which means that people have different understandings of childhood abuse and neglect in different cultural backgrounds. The understanding of neglect is different between cultures, which limits the generalizability of the results.

## Conclusion

In this study, psychological abuse and neglect were positively correlated with the mental problems of college students during the COVID-19 pandemic, and the experience of reproving increased the risk of psychological problems. This study allows us to better understand the relationship between child abuse and mental health during stressful events, which may facilitate the development of stressful event intervention programs.

## Data Availability Statement

The original contributions presented in the study are included in the article/Supplementary Material, further inquiries can be directed to the corresponding author/s.

## Ethics Statement

The studies involving human participants were reviewed and approved by School of Exercise and Health Sciences, Xi'an Physical Education University. Written informed consent from the participants's legal guardin/next of kin was not required for this study in accordance with the national legislation and the institutional requirements.

## Author Contributions

ZZ designed this survey, commented and revised the manuscript, and wrote the final version. PL contributed to recruiting participants, data collection, and writing the initial draft. LH were responsible for data analysis. All authors contributed to the final draft of the manuscript.

## Conflict of Interest

The authors declare that the research was conducted in the absence of any commercial or financial relationships that could be construed as a potential conflict of interest.

## Publisher's Note

All claims expressed in this article are solely those of the authors and do not necessarily represent those of their affiliated organizations, or those of the publisher, the editors and the reviewers. Any product that may be evaluated in this article, or claim that may be made by its manufacturer, is not guaranteed or endorsed by the publisher.
