# Supplementary material for: Correlation of Childhood Psychological Abuse and Neglect With Mental Health in Chinese College Students During the COVID-19 Pandemic
Source: Front Psychiatry. 2022 Jan 5;12:770201. doi: 10.3389/fpsyt.2021.770201 (PMC8766813; doi:10.3389/fpsyt.2021.770201)
Supplement: Supplementary file 1 [file Table_1.docx]

**Supplementary Table 1. Comparison of the SRQ-20 scores of students with or without psychological abuse/neglect**

| Variable |  | N | Mean | SD | t | *p* |
| --- | --- | --- | --- | --- | --- | --- |
| Psychological abuse | Without psychological abuse | 80 | 4.23 | 4.595 | -5.214 | <0.01 |
|  | With psychological abuse | 101 | 8.21 | 5.472 |  |  |
| Psychological neglect | With psychological neglect | 86 | 4.10 | 4.522 | -6.000 | <0.01 |
|  | Without psychological neglect | 95 | 8.57 | 5.392 |  |  |
