# Supplementary material for: Correlation of Childhood Psychological Abuse and Neglect With Mental Health in Chinese College Students During the COVID-19 Pandemic
Source: Front Psychiatry. 2022 Jan 5;12:770201. doi: 10.3389/fpsyt.2021.770201 (PMC8766813; doi:10.3389/fpsyt.2021.770201)
Supplement: Supplementary file 2 [file Table_2.docx]

**Supplementary Table2： The correlation coefficient between the independent variables**

|  |  | **reproving** | **intimidation** | **interference** | **emotional neglect** | **educational neglect** | **physical neglect** |
| --- | --- | --- | --- | --- | --- | --- | --- |
| reproving | Pearson | 1 | 0.82^**^ | 0.69^**^ | 0.72^**^ | 0.62^**^ | 0.59^**^ |
|  | *p* |  | <0.01 | <0.01 | <0.01 | <0.01 | <0.01 |
| **intimidation** | Pearson | 0.82^**^ | 1 | 0.63^**^ | 0.69^**^ | 0.58^**^ | 0.52^**^ |
|  | *p* | <0.01 |  | <0.01 | <0.01 | <0.01 | <0.01 |
| **interference** | Pearson | 0.69^**^ | 0.63^**^ | 1 | 0.53^**^ | 0.49^**^ | 0.40^**^ |
|  | *p* | <0.01 | <0.01 |  | <0.01 | <0.01 | <0.01 |
| emotional neglect | Pearson | 0.72^**^ | 0.69^**^ | 0.53^**^ | 1 | 0.82^**^ | 0.77^**^ |
|  | *p* | <0.01 | <0.01 | <0.01 |  | <0.01 | <0.01 |
| **educational neglect** | Pearson | 0.62^**^ | 0.58^**^ | 0.49^**^ | 0.82^**^ | 1 | 0.71^**^ |
|  | *p* | <0.01 | <0.01 | <0.01 | <0.01 |  | <0.01 |
| **physical neglect** | Pearson | 0.59^**^ | 0.52^**^ | 0.40^**^ | 0.77^**^ | 0.71^**^ | 1 |
|  | *p* | <0.01 | <0.01 | <0.01 | <0.01 | <0.01 |  |
