# Supplementary material for: Correlation of Childhood Psychological Abuse and Neglect With Mental Health in Chinese College Students During the COVID-19 Pandemic
Source: Front Psychiatry. 2022 Jan 5;12:770201. doi: 10.3389/fpsyt.2021.770201 (PMC8766813; doi:10.3389/fpsyt.2021.770201)
Supplement: Supplementary file 3 [file Table_3.docx]

**Supplementary Table3：Multicollinearity Analysis**

| model | B | SB | t | *p* | TOL | VIF |
| --- | --- | --- | --- | --- | --- | --- |
| reproving | 0.15 | 0.07 | 2.32 | 0.02 | 0.25 | 4.07 |
| intimidation | 0.07 | 0.07 | 1.01 | 0.29 | 0.30 | 3.32 |
| interference | -0.01 | 0.06 | -0.11 | 0.91 | 0.51 | 1.96 |
| emotional neglect | 0.06 | 0.10 | 0.65 | 0.52 | 0.21 | 4.84 |
| educational neglect | 0.03 | 0.06 | 0.49 | 0.62 | 0.31 | 3.26 |
| physical neglect | -0.04 | 0.06 | -0.59 | 0.55 | 0.38 | 2.61 |
| (constant) | 0.08 | 0.07 | 1.23 | 0.22 |  |  |
